# Ketoproof? A systematic classification of pharmaceutical excipients for ketogenic diet therapy

**DOI:** 10.1007/s11096-026-02156-6

**Published:** 2026-05-05

**Authors:** Samir El Abdouni, Manon G. den Uil, Zikrullah Kafa, Tatiana Soares Ribeiro, Elles J. T. M. van der Louw, Elisabeth J. Ruijgrok, A. Laura Nijstad

**Affiliations:** 1 Department of Hospital Pharmacy, Erasmus MC, University Medical Center, Doctor Molewaterplein 40, 3015GD Rotterdam, The Netherlands; 2 Division of Dietetics, Department of Internal Medicine, Erasmus MC, Rotterdam, The Netherlands

**Keywords:** Clinical, Decision support systems, Diet, Drug resistant epilepsy, Excipients, Ketogenic

## Abstract

**Introduction:**

Ketogenic diet therapy (KDT) is an evidence-based treatment for refractory epilepsy, particularly in children. Strict adherence to carbohydrate restriction is essential to maintain ketosis, yet many medications contain excipients that can disrupt this balance. Pharmacists play a crucial role in evaluating and adjusting medication regimens for patients on KDT, but limited transparency regarding excipient content often makes this process inefficient and inconsistent. Although some regional databases exist, no systematic and comprehensive resources have been developed for broader use.

**Aim:**

This study aimed to systematically classify pharmaceutical excipients and label all medications marketed in the Netherlands according to their compatibility with KDT (“Ketoproof” status) using a transparent, reproducible framework that can serve as the foundation for safer prescribing and international standardization.

**Method:**

A retrospective cross-sectional database analysis was conducted using data from the Dutch G-Standaard (June 2023–March 2024), a national pharmaceutical reference database used in the Netherlands. All excipients authorized in medications marketed in the Netherlands were extracted and classified based on chemical composition, metabolic fate, and administration route. Excipients were assigned a Ketoproof status through a structured decision flowchart, expert consensus, and quality control review. Finally, pharmaceutical products were labeled by cross-referencing their excipient profiles against the classified dataset.

**Results:**

A total of 1047 excipients were identified. Of these, 778 (74%) were successfully classified: 590 (56%) as Ketoproof and 188 (18%) as non-Ketoproof. The remaining 269 (26%) were unclassified. Excipients requiring expert consensus included polyols and sugar alcohols, organic acids, and coloring and flavoring agents, which were carefully assessed for clinical relevance. Among 28,721 pharmaceutical products analyzed, 41% (n = 11,781) were labeled as Ketoproof. The full dataset was integrated into KetoMed, a web-based tool designed to support healthcare professionals in reviewing medications for patients on KDT.

**Conclusion:**

This study presents the first systematic classification of pharmaceutical excipients for KDT compatibility. The transparent, evidence-informed framework enables structured labeling of medications and supports safer prescribing for patients undergoing KDT. Broader adoption and international collaboration are recommended to establish consensus on excipient classification, enhance regulatory transparency, and integrate Ketoproof labeling into clinical decision-support systems to optimize ketogenic therapy management.

**Supplementary Information:**

The online version contains supplementary material available at https://doi.org/10.1007/s11096-026-02156-6.

## Impact statements

- The Ketoproof classification framework enables clinical pharmacists to rapidly identify safer medication alternatives for patients on KDT, reducing the risk of unintended dietary disruption.
- The methodology is adaptable to other national drug markets and can be integrated into clinical decision-support systems.
- The findings underscore the need for greater regulatory transparency regarding pharmaceutical excipient composition and provide a methodological precedent for advocating selective excipient disclosure.

## Introduction

Ketogenic diet therapy (KDT) is an evidence-based treatment for medication-resistant epilepsy, particularly in pediatric patients, where it contributes to seizure control [1]. This population frequently experiences difficulties swallowing solid oral dosage forms, which increases reliance on liquid formulations. Such formulations commonly contain carbohydrate‑based excipients added for palatability or stability, which may compromise the stringent carbohydrate restrictions required to maintain therapeutic ketosis [2].

KDT consists of a high-fat, low-carbohydrate diet that mimics the metabolic effects of fasting. Under normal conditions, glucose serves as the body’s primary energy source, entering cells to be metabolized through glycolysis, the tricarboxylic acid (TCA) cycle (also known as the Krebs cycle), and oxidative phosphorylation to produce adenosine triphosphate (ATP). In restricting carbohydrate intake, glucose availability drops, and the body shifts to fatty acid oxidation as its primary energy source. Fatty acids undergo β-oxidation in the liver, generating acetyl-CoA, which would typically enter the TCA cycle by combining with oxaloacetate. However, during fasting or carbohydrate restriction, oxaloacetate is increasingly diverted toward gluconeogenesis, reducing its availability for the TCA cycle. As a result, excess acetyl-CoA is redirected toward ketogenesis, yielding ketone bodies: acetoacetate and β-hydroxybutyrate, and acetone [3]. These ketone bodies become the main fuel source for peripheral tissues, including the brain [3].

Achieving and maintaining ketosis is demanding. The ketogenic diet ratio, the ratio of grams of fat to the sum of grams of protein and carbohydrates, is a key component of therapy. Even one gram of additional carbohydrate can significantly disrupt the metabolic state of ketosis, which is required for therapeutic efficacy. Notably, in young children, where a 3:1 ratio is often recommended in accordance with clinical guidelines for infants with refractory epilepsy [4]. The state of ketosis can be disrupted by medication, which often contains carbohydrate excipients in the form of sugars, sugar alcohols, and starches [5–9]. Several strategies have been proposed to minimize carbohydrate exposure from medication. One longstanding, yet outdated strategy was to co-administer a small amount of butter or oil with carbohydrate-containing medications to compensate metabolically. Although this strategy may theoretically compensate for the carbohydrate contribution of medication, its practical applicability is limited, particularly for treatments requiring multiple daily. An alternative approach involves slightly increasing the overall ketogenic ratio to account for the estimated carbohydrate content in medication throughout the day [10]. However, this can result in overly strict dietary prescriptions, making adherence to KDT more challenging, particularly for young children and their families. In our clinical practice, the preferred strategy is to substitute high-carbohydrate-content liquid medication for lower carbohydrate alternatives or solid formulations whenever possible [2, 5].

At our center, KDT is delivered through a multidisciplinary approach involving physicians, pharmacists, and dieticians. Specifically, when a patient starts KDT, a pharmacist reviews the complete medication list and proposes changes to limit carbohydrate intake, ideally to no more than one gram of absorbable carbohydrates per day [5, 7]. If a medication contains carbohydrates and no suitable alternative with the same active ingredient is available, a pharmacotherapeutic equivalent is considered. When no suitable substitution exists, the pharmacist consults the dietitian to adjust the ketogenic ratio to accommodate the additional carbohydrate load. To calculate the daily intake of carbohydrates from medication, pharmacists require quantitative data on the excipient composition, which is often not disclosed by manufacturers [11, 12]. When such data are not available, pharmacists must request information directly from manufacturers—a process that is labor‑intensive, inefficient, and often yields inconsistent or outdated data. Moreover, locally registered information requested in the past may no longer reflect current formulations, as manufacturing processes and ingredient compositions evolve over time. This manual effort diverts pharmacists’ time from more impactful clinical tasks and underscores the need for a more structured, transparent, and sustainable approach. Although relevant data exist, they are typically fragmented, inaccessible to clinicians, or presented in a format unsuitable for structured clinical decision-making.

To our knowledge, there is currently only one publicly available online database addressing this issue: KetoDietCalculator. This is regularly updated and includes information on over 600 medications commonly used in children with refractory epilepsy in the United States [13]. In another such initiative, Martín Prado et al*.* reported the carbohydrate content, expressed in caloric value, of 400 medications frequently used in pediatric neurology in Spain [14]. However, no systematically developed or broadly applicable resource exists for many other healthcare systems, including those in Europe, where drug formulations may differ substantially between markets.

### Aim

The aim of this study was to systematically classify pharmaceutical excipients and label medicines according to their compatibility with KDT. We sought to establish a reproducible framework that enables safer prescribing and can serve as a foundation for international standardization. Specifically, this study aimed to develop an overview of all pharmaceutical products marketed in the Netherlands and assign each a “Ketoproof” status based on excipient compatibility.

## Method

### Study design

We conducted a retrospective cross-sectional database analysis to assess the compatibility of pharmaceutical excipients with the therapeutic KDT.

### Data source

We extracted a list of pharmaceutical excipients from the Dutch ‘G-standaard’ database, [15] which covers all excipients authorized in medications marketed in the Netherlands. For each pharmaceutical product, this dataset contained: the active component, the excipients, and the registered administration routes.

### Classification and labeling

**Fig. 1:**
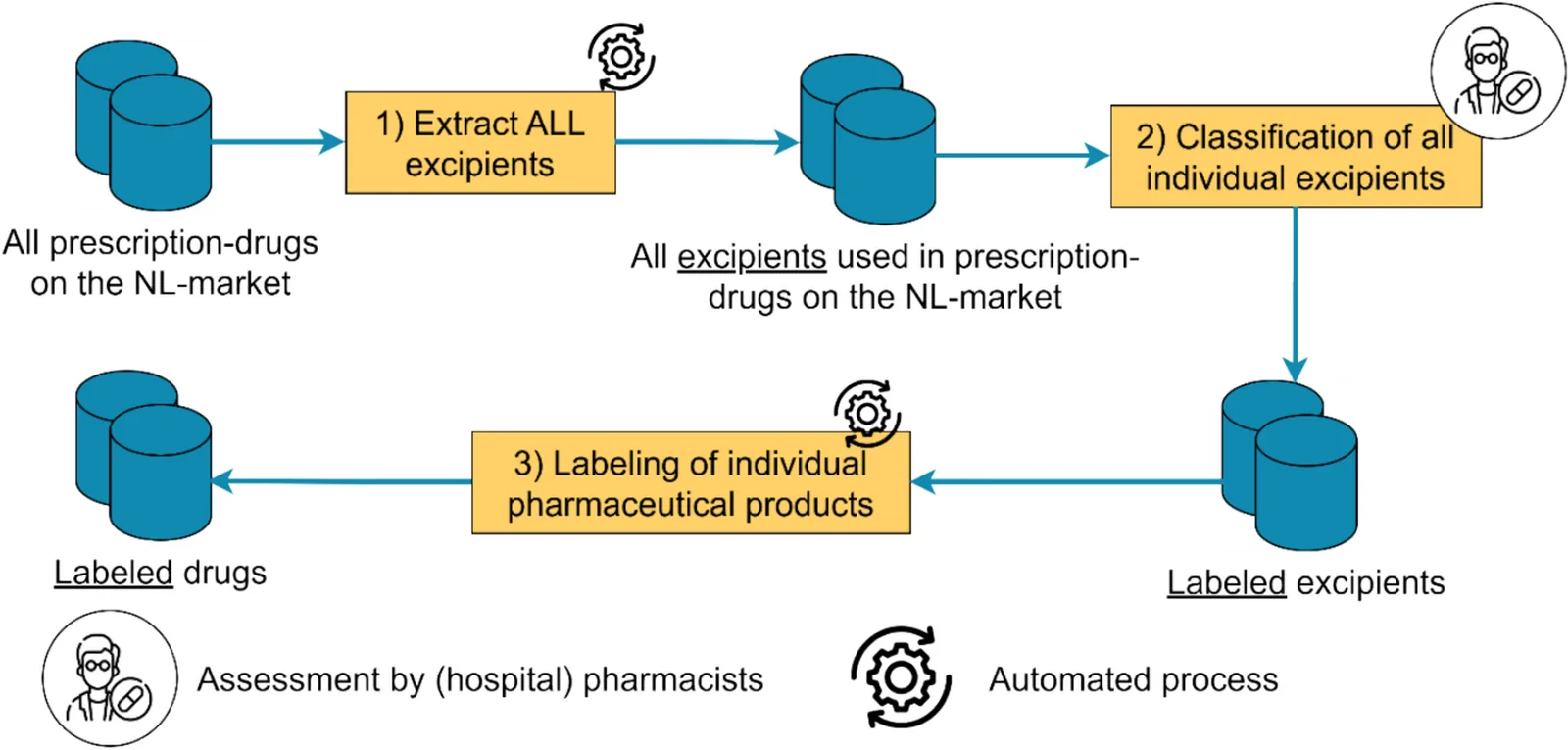
Schematic overview of the classification and labeling process. Labeled excipients refers to assignment of Ketoproof status (yes/no) to each excipient; Labeled drugs refers to assignment of Ketoproof status (yes/no) to each pharmaceutical product based on its excipient profile

**Fig. 2:**
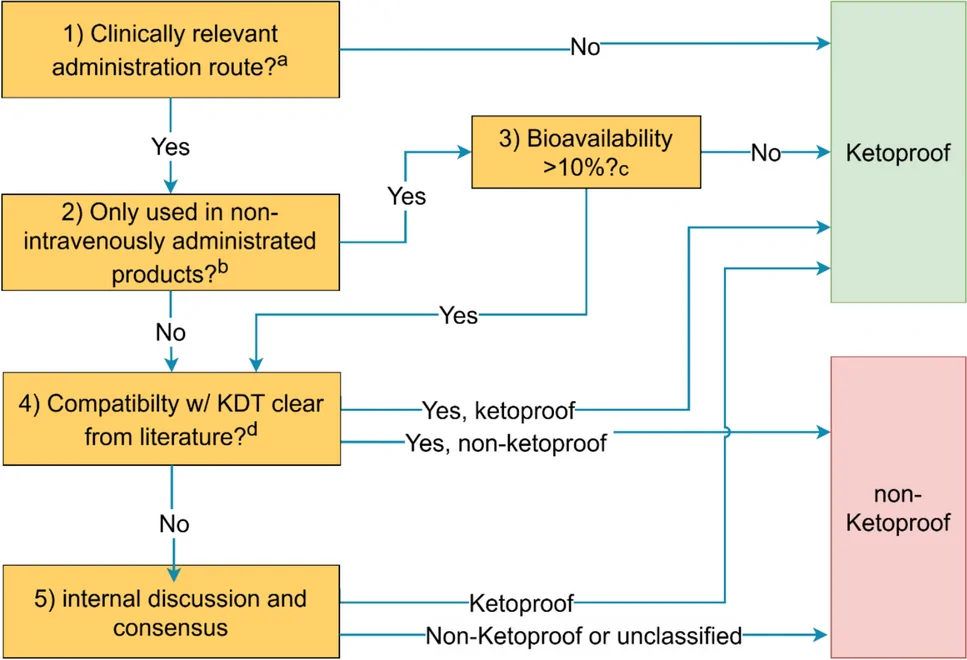
Excipient classification flowchart. ^a^As defined in Supplementary Table 1. ^b^I.e., if an excipient is only used in orally administered products. ^c^The highest value found in literature was applied. ^d^Literature data were obtained from PubMed, PubChem, and regulatory databases (EMA, FDA), with unclear cases resolved through expert consensus discussion (step 5) based on chemical composition, functional role, administration route and metabolic fate. *KDT* ketogenic diet therapy

We developed a systematic approach for classifying excipients and labeling pharmaceutical products based on their compatibility with KDT. This process was applied in the period of June 2023 to March 2024 and consisted of two steps: (1) classification of excipients, (2) labeling of pharmaceutical products as ‘*Ketoproof*’ yes or no? (see Fig. 1). The classification of all individual excipients is depicted in more detail in Fig. 2.

The classification and labeling process was carried out by (hospital) pharmacists over six months, with detailed records kept for each classification, including the responsible assessor, the date, and the references used. All participating pharmacists attended a briefing session in which the classification criteria, the decision flowchart, and documentation procedures were reviewed.

### Excipient prioritization

To focus our excipient-classification efforts on the most clinically relevant pharmaceutical products, we prioritized on two levels: (1) by administration route and (2) by active substance.

We extracted the distinct administration routes from the dataset, which we then mapped to a priority score on a 0–3 scale: 3 = High: Routes where precise carbohydrate control is most critical (e.g. intravenous because of a bioavailability of 100%); 2 = Medium–high: substantial systemic absorption is expected, but less acute than parenteral (e.g. oral); 1 = Low: primarily local effect or minimal systemic absorption (e.g. ocular); 0 = Not relevant: excipient carbohydrate content has negligible clinical impact (e.g. cutaneous cream). These priority levels were determined through internal discussions relevance and consensus regarding the clinical among the involved (hospital) pharmacists on the research team.

Finally, we assigned the highest applicable priority score to each pharmaceutical product. Please refer to Supplementary Table 1 for an overview of each administration route and its priority score.

In parallel, we curated a list of active substances considered most relevant by the (hospital) pharmacists in our research team based on usage data from our clinical practice (see Supplementary Table 2). We then cross-referenced this list against every excipient occurrence: any excipient present in a formulation containing at least one of those high-priority actives was assigned a “high” prioritization score (1), and all others were assigned “low” (0). This list functioned as a prioritization tool to direct initial classification efforts toward excipients occurring in high-priority products; it did not constitute an exclusion criterion.

### Excipient classification

Each excipient was systematically classified based on its chemical composition, functional role, administration route, bioavailability, and its role in relevant metabolic pathways. This process was visualized in a decision flowchart (see Fig. 2) and guided the assignment of the *Ketoproof*-status of each excipient.

To ensure internal consistency and adherence to the predefined classification criteria, the results were independently reviewed by two members of the research team. In addition, a random sample comprising 50% of all classifications was re-evaluated by a second reviewer as a quality control measure. This threshold was chosen as a pragmatic balance between thorough verification and feasibility given the scale of the dataset. For excipients with unclear *Ketoproof* status, classification decisions were made through internal expert discussion and consensus.

### Pharmaceutical product labeling

We automated the labeling of pharmaceutical products (i.e., medications) for ketogenic compatibility using a custom Python script (Python Software Foundation, Python Language Reference, version 3.10.4, Delaware, United States). This script merged datasets from the Dutch G-Standaard database to compile excipient profiles for each product and then cross-referenced them against our dataset of classified excipients. Medications were labeled as Ketoproof (‘Yes’) if all their excipients were classified as Ketoproof, or as non-Ketoproof (‘No’) if one or more excipients were classified as non-Ketoproof or remained unclassified. The results of the automated labeling were manually reviewed by a research team member using a random sample of 50 labeled medications. The script was developed by a member of the research team (SEA) with training in programming and data analysis. The script and its documentation are available upon reasonable request.

The labeled dataset was implemented in a custom-built web application (KetoMed), specifically designed to support healthcare professionals in reviewing medication for patients on a KDT. At the time of writing, the application is accessible to authorized users as part of a pilot study, the results of which will be presented as a separate study.

### Ethics approval

No ethics approval was required for this study.

## Results

### Excipient classification

**Fig. 3:**
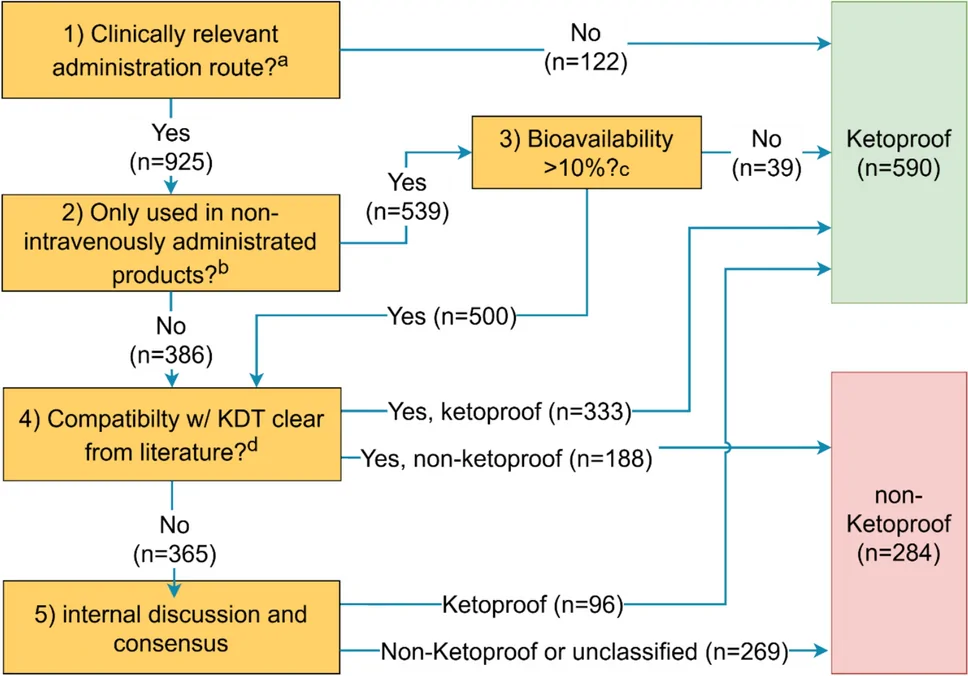
Excipient classification results. ^a^As defined in Supplementary Table 1. ^b^I.e., if an excipient is only used in orally administered products. ^c^The highest value found in literature was applied. ^d^Literature data were obtained from PubMed, PubChem, and regulatory databases (EMA, FDA), with unclear cases resolved through expert consensus review. *KDT*   ketogenic diet therapy

Of the 1047 excipients found in the dataset, 150 (14%) and 236 (23%) were prioritized as “high” and “medium–high”, respectively. Of the total excipients, 778 (74%) excipients were successfully classified. Out of all available excipients, 590 (56%) were *Ketoproof*, 188 (18%) *non-Ketoproof*, and 269 (26%) unclassified. A more detailed overview of the classification is presented in Fig. 3.

An excerpt of the dataset is presented in Supplementary 3. The full, up—to-date, dataset can be accessed upon request.

### Internal discussion and consensus

The 365 excipients where classification could not be established based on literature alone, were discussed with the purpose of classifying. This resulted in 96 excipients classified as Ketoproof and 269 excipients classified as non-Ketoproof. Compounds that prompted discussion included polyols and (sugar) alcohols, organic acids, and coloring and flavoring agents. Similarly, excipients only used in single administrations—such as vaccines—were discussed and classified as Ketoproof.

### Labeling

Out of the 28,721 individual pharmaceutical products, 41% (n = 11,781) were labeled as *Ketoproof*. An illustrative example of the labeled products is presented in Table 1.

**Table 1:** Illustrative examples of labeled pharmaceutical products, translated to English

Pharmaceutical product	Ketoproof?	Non-ketoproof excipients
Amoxicillin Dispersible Actavis Tablet 750 mg	Yes	–
Amoxicillin Sandoz Powder for Suspension 250 mg/5 mL	No	Sodium benzoate; guar gum; benzyl alcohol
Clobazam Capsule 2.5 mg CEB	Yes	–
Clobazam Suspension 1 mg/mL CEB	No	Methylparaben; propylparaben; sucrose
Depakine Liquid for children 300mg/ml (Valproic acid)	Yes	–
Depakine Sugarfree Syrup 40mg/ml (Valproic acid)	No	Methyl parahydroxybenzoate sodium; Propyl parahydroxybenzoate sodium; Sorbitol
Lacosamide Accord Film-Coated Tablet 150 mg	Yes	–
Lacosamide Xiromed Syrup 10 mg/mL	No	Glycerol; methylparaben; sorbitol; maltol; propylene glycol

Manual review of a random sample of 50 labeled pharmaceutical products confirmed full concordance with the automated labeling output; no discrepancies were identified.

The full, up—to-date dataset can be accessed upon request through: https://ketomed.nl.

### Ketoproof rate by administration route

Of the 28,722 pharmaceutical products analyzed, the Ketoproof rate varied considerably by administration route, as presented in Table 2. Parenteral products showed the highest Ketoproof rate at 60% (n = 4766 of 7971). Oral products—the largest category with 13,751 products—had a markedly lower Ketoproof rate of 22% (n = 2995).

**Table 2:** Ketoproof status of pharmaceutical products marketed in the Netherlands by administration route

Administration route	Ketoproof (n)	Not Ketoproof (n)	Total (n)	Ketoproof rate (%)
Oral	2995	10,756	13,751	22%
Parenteral	4766	3205	7971	60%
Cutaneous	704	1379	2083	34%
Ocular	474	218	692	68%
Rectal	355	305	660	54%
Inhalation	225	199	424	53%
Nasal	161	134	295	55%
Transdermal	143	34	177	81%
Total	9823	16,230	26,053	38%

For each route, products were labeled Ketoproof (yes) or not Ketoproof (no) based on cross-referencing their excipient profiles against the classified excipient dataset.

### Most common non-Ketoproof excipients

**Fig. 4:**
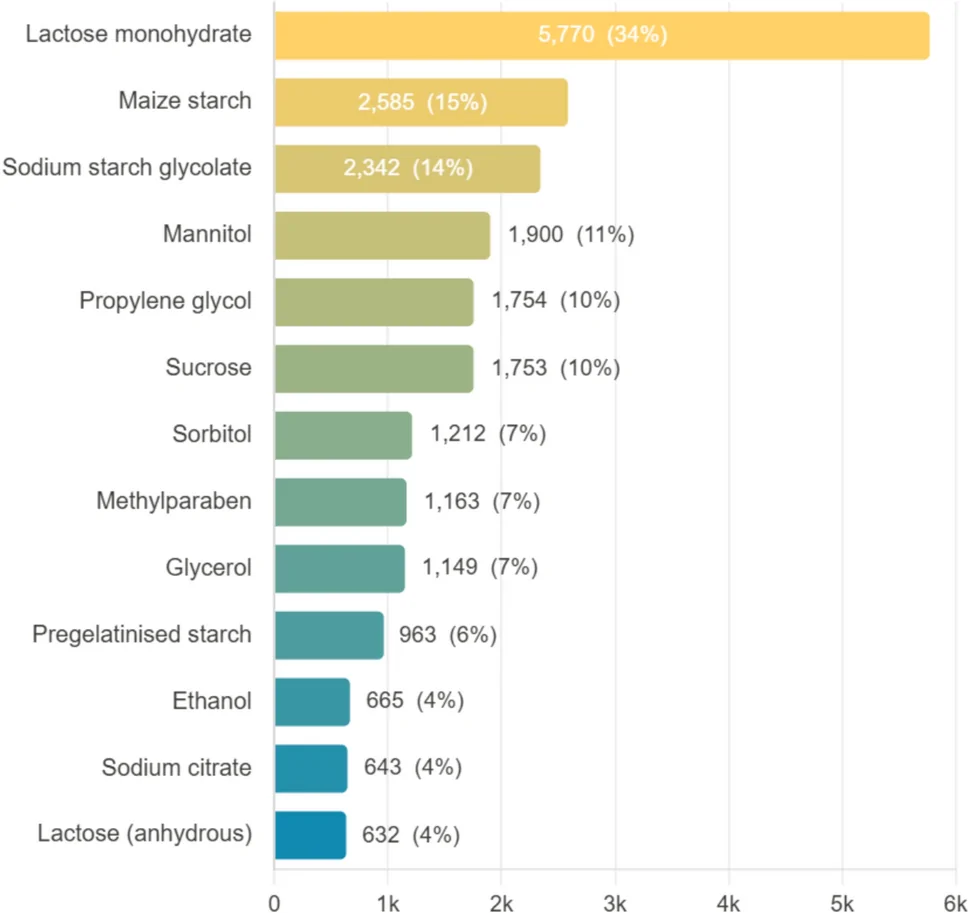
Most frequently occurring non-Ketoproof excipients across pharmaceutical products marketed in the Netherlands. Bars represent the number of products containing each excipient, expressed as absolute count and as a percentage of all non-Ketoproof products (n = 16,928). Only excipients classified as non-Ketoproof are included; products may contain more than one non-Ketoproof excipient

Across the 16,928 non-Ketoproof products, lactose monohydrate was by far the most frequently occurring non-Ketoproof excipient, present in 5770 product formulations, followed by maize starch (n = 2585) and sodium starch glycolate (n = 2342). These three excipients together accounted for the majority of non-Ketoproof classifications. An overview of the top 10 most commonly used non-Ketoproof excipients is presented in Fig. 4.

## Discussion

This study is the first to systematically classify pharmaceutical products classify pharmaceutical excipients for KDT compatibility—and subsequently pharmaceutical products—based on the presence or absence of non-ketoproof excipients. By applying a structured, evidence-informed approach, we addressed a long-standing need for a standardized method to support clinical decision-making in this context.

Out of the 1047 excipients, 778 were successfully categorized. The remaining excipients were left unclassified and defaulted to *non-Ketoproof*. Throughout the classification process, several consensus decisions were necessary. Organic acids, for example, emerged as a debatable group due to their direct entry into the TCA-cycle, potentially undermining the therapeutic mechanism of ketosis. Their metabolism could possibly reduce the drive towards ketosis, and through anaplerotic replenishment of oxaloacetate, they may also promote gluconeogenesis. However, organic acids are typically used in such small quantities that they are not expected to have a clinically significant effect and were therefore classified as *Ketoproof*. Similarly, alcohols such as propylene glycol and benzyl alcohol are metabolized into organic acids that fuel the TCA-cycle [16]. In contrast to organic acids, alcohols are expected to exert a clinically significant effect on ketosis, primarily due to the substantially higher quantities in which they are typically administered. This is supported by the fact that propylene glycol is used to reverse ketosis in animals, which further illustrates its incompatibility with KDT [17]. This effect is due to increased oxidation of acetyl-CoA and enhanced glucose production via gluconeogenesis. The resulting rise in insulin secretion leads to reduced circulating fatty acid concentrations, which are the primary substrate for ketogenesis [18, 19]. Ethanol, while not affecting the TCA-cycle, also poses risks by altering glucose metabolism [20, 21]. In addition, ethanol can lead to hypoglycemia, and if the administration of sugar or glucose becomes necessary, ketosis may be disrupted, potentially resulting in epileptic seizures [22]. These observations underline the importance of considering not only the chemical class of excipients but also their metabolic fate. Therefore, we have decided to classify propylene glycol, benzyl alcohol, and ethanol as *non-Ketoproof*.

In contrast, flavoring and coloring agents were assumed to be *Ketoproof*, given their minimal quantities in formulations, despite the lack of detailed compositional data. Excipients with a maximum bioavailability of 10% were classified as compatible, based on the assumption that such low systemic availability has no clinically relevant impact on ketogenic diet therapy. Furthermore, we identified excipients exclusively used in pharmaceutical formulations intended for single administration, such as vaccines. These were similarly considered *Ketoproof*, as single exposure is unlikely to have a clinically relevant impact and often no alternatives are available. These assumptions were necessary due to limitations in available information.

The strength of our approach lies in its systematic design, using the national medication database and structured documentation of each classification. This level of traceability ensures transparency and allows for regular updates as new evidence becomes available. The resulting classification has been implemented in a web-based tool, which is, at the time of writing, being piloted in clinical practice.

However, several limitations must be acknowledged. Firstly, our labeling is based on pharmaceutical products marketed in the Netherlands and may not be directly generalizable to other countries due to differences in formulation. Nevertheless, the methodology and decision framework are broadly applicable and could be adapted to other regulatory environments. Secondly, there is currently no formal consensus among Dutch expertise centers regarding the classification of some excipients, such as artificial sweeteners and alcohols. This would be required for broad implementation of structural decision-support solutions. Finally, the limited availability of quantitative composition data for excipients, which is typically not disclosed by manufacturers, is not solved by our approach.

To support the usability of excipient classification for KDT, we advocate for collaboration among KDT-experts in order to reach consensus and establish standardized definitions and thresholds for excipient compatibility. We also call on regulatory agencies and industry stakeholders to promote transparency in pharmaceutical excipient content, if relevant for KDT. Existing precedents, such as mandatory labeling of ethanol, lactose, and propylene glycol, demonstrate that selective disclosure is feasible and impactful.

## Conclusion

This study demonstrates that systematic, evidence-informed classification of pharmaceutical excipients for KDT compatibility is feasible at national scale. These findings provide a concrete foundation for future international collaboration, consensus development on excipient thresholds, and integration of Ketoproof labeling into clinical decision-support systems, ultimately supporting safer prescribing for patients on KDT.

## Supplementary Information

Below is the link to the electronic supplementary material.

Supplementary file1 (DOCX 34 kb)

## Data Availability

Data can be requested upon reasonable request.
