# Supplementary material for: Ketoproof? A systematic classification of pharmaceutical excipients for ketogenic diet therapy
Source: Int J Clin Pharm. 2026 May 5;48(5):1850–7. doi: 10.1007/s11096-026-02156-6 (PMC13569564; doi:10.1007/s11096-026-02156-6)
Supplement: Supplementary file 1 [file 11096_2026_2156_MOESM1_ESM.docx]

Supplementary Information

**Title**: Ketoproof? A Systematic Classification of Pharmaceutical Excipients for Ketogenic Diet Therapy

**Short title**: Ketoproof? Excipients & KDT

**Authors:** Samir El Abdouni, Manon G. den Uil, Zikrullah Kafa, Tatiana Soares Ribeiro, Elles J.T.M. van der Louw, Elisabeth J. Ruijgrok, A. Laura Nijstad

**Corresponding investigator:** A. Laura Nijstad ([a.nijstad@erasmusmc.nl](mailto:a.nijstad@erasmusmc.nl))

Supplementary Information Table 1 - Administration routes by excipient-classification priority-scores

| Priority | | | |
| --- | --- | --- | --- |
| High | **Medium-high** | **Low** | **Not relevant** |
| Parenteral | Oral | Inhalation | Cutaneous |
| Intravenous | Sublingual | Ocular | Transdermal |
| Subcutaneous | Oromucosal | Rectal | Local |
| Intravesical | Intrathecal | Nasal | Intratympanal |
| Intramuscular |  | Intra-uterine | Intratumoral |
| Intralesional |  | Vaginal | Urethral |
| Intraperitoneal |  | Intravitreal | Endocervical |
| Hemodialysis |  | Intradermal | Extracorporal |
| Intra-articular |  | Auricular | Intracameral |
| Epilesional |  | Dental |  |
| Intrapleural |  | Endotracheopulmonal |  |
| Intra-arterial |  | Gastro-enteral |  |
|  |  | Epidural |  |
|  |  | subretinal |  |

Priority levels were assigned based on internal expert consensus, reflecting the expected systemic exposure and clinical relevance of each route in the context of ketogenic diet therapy.

Supplementary Information Table 2 - Prioritized active substances

| Active substances | | |
| --- | --- | --- |
| Alimemazine | Fluticasone | Paracetamol |
| Alprazolam | Gabapentin | Perampanel |
| Amoxicillin | Glucose/sodium chloride | Phenytoin |
| Azithromycin | Glycopyrronium | Potassium citrate |
| Baclofen | Granisetron | Prednisolone |
| Brivaracetam | Hydrocortisone | Pregabalin |
| Calcium carb/colecal | Lacosamide | Primidone |
| Carbamazepine | Lamotrigine | Risperidone |
| Cefuroxime | Levetiracetam | Risperidone drink |
| Chloral hydrate | Levocarnitine | Rufinamide |
| Clobazam | Levothyroxine | Salbutamol |
| Clonazepam | Lorazepam | Sodium chloride |
| Cooling ointment | Macrogol | Stiripentol |
| Cotrimoxazole | Macrogol/salts | Sultiam |
| Davitamon vitamin d | Melatonin | Taurine |
| Diazepam | Midazolam | Temazepam |
| Diclofenac | Mometasone | Thiamazole |
| Docusic acid/sorbitol | Nadroparine | Topiramate |
| Esomeprazole | Nitrazepam | Valproic acid |
| Ethinyl estradiol/levonorgestrel | Omeprazole | Vigabatrin |
| Ethosuximide | Oseltamivir | Zinc oxide |
| Felbamate | Oxazepam | Zonisamide |
| Fenobarbital | Oxcarbazepine |  |

The active substances listed were selected based on usage data from our clinical center, reflecting the most commonly prescribed medications in pediatric patients undergoing ketogenic diet therapy.

Supplementary Information Table 3 - Random excerpt of the classified excipient dataset

| Excipient (English) | Only in non-IV administered products? | Non-IV administration routes | If orally administered: bioavailability | Ketoproof? |
| --- | --- | --- | --- | --- |
| Sesame oil | No |  |  | Yes |
| Water, purified (demineralized) | No |  |  | Yes |
| Colophonium | No |  |  | Unclassified |
| Glycol salicylate | No |  |  | Yes |
| Azorubine | No |  | <10% | Yes |
| Grape essence | No |  |  | Yes |
| Daratumumab 1800 mg | No |  |  | Yes |
| Carbomer, species unknown | Yes | Ocular, transdermal, rectal, oral, cutaneous | 0% | Yes |
| Peppermint essence | No |  |  | Yes |
| Copper | No |  |  | Unclassified |
| Polyisobutene | No |  |  | Yes |
| Lauramine oxide | No |  |  | Yes |
| Mandelic acid | No |  |  | No |
| Urea | No |  |  | Unclassified |
| Octyl gallate | No |  |  | Unclassified |
| Propylene glycol monocaprylate | No | Oral, ocular, rectal | None | No |
| Carbomer 934P | Yes |  | 0% | Yes |
| Tin(II) chloride 2-water | No |  |  | Unclassified |
| Polyvinyl chloride | No |  |  | Unclassified |
| Lavender oil | No |  |  | Yes |
| Glutamic acid | No |  |  | Yes |
| Starch, type unknown, gluten-free | No |  |  | No |
| Apricot essence | No |  |  | Yes |
| Tocofersolan | No |  |  | Unclassified |
| Ethanol, denatured | No |  |  | No |
